# Treatment of the small saphenous vein and tributary veins with endolaser associated with ultrasound-guided foam in a patient with post-thrombotic syndrome: presenting the TEThA technique

**DOI:** 10.1590/1677-5449.202301422

**Published:** 2024-11-29

**Authors:** Nara Medeiros Cunha de Melo Vasconcelos, Harue Santiago Kumakura, Marcelo Halfen Grill, Marília de Castro e Silva

**Affiliations:** 1 Hospital Dia Anjos Vasculares, Natal, RN, Brasil.; 2 Centro de Estudos Hiroshi Miyake, São Paulo, SP, Brasil.

**Keywords:** varicose veins, saphenous vein, chronic venous insufficiency, endovenous laser therapy, esthetics

## Abstract

Chronic venous disease of the lower limbs is a highly prevalent pathology and endovenous thermoablation is the technique of choice for treatment of insufficient saphenous veins. However, there is still controversy about the best management for varicose tributaries. This article reports a case of outpatient treatment of reflux of the small saphenous vein and tributary veins in a 52-year-old female patient with post-thrombotic syndrome complaining of pain and edema in the right lower limb. We performed the Transfixing Endovenous Thermal Ablation (TEThA) technique with thermoablation of the small saphenous vein and varicose veins combined with ultrasound-guided administration of 2% polidocanol foam. After 30 days, the control Doppler ultrasound showed occlusion of the short saphenous vein and absence of ultrasound signs of varicose veins and thrombosis. The combined endovenous and perivenous treatment of lower limb varicose veins proved to be safe, fast, and effective.

## INTRODUCTION

Chronic venous disease (CVD) of the lower limbs (LL) is a highly prevalent pathology.^1^ If left untreated, 22% of patients classified as CEAP C2^2^ will progress to venous ulcers within 6 years.^1^ Postthrombotic syndrome (PTS) is a constellation of signs and symptoms of CVD caused by impaired venous flow because of deep venous obstruction and/or reflux after deep venous thrombosis. Typical signs are pain, varicose veins, edema, and cutaneous changes, including venous ulcers, involving the LL. This syndrome occurs in 20 to 50% of patients with DVT, 5 to 10% of whom develop severe PTS.^3^

While endovenous thermoablation is considered the technique of choice for treatment of incompetent saphenous veins, there is still controversy with relation to the best form of management for varicose tributaries (VTs).^4^ Although concurrent treatment of VTs, using phlebectomy, is still indicated,^4^ there is a tendency to seek less invasive techniques, such as chemical or thermal sclerotherapy, and even to wait until after ablation of the incompetent saphenous vein, to observe the repercussions of reduced venous pressure for remodeling of VTs, before deciding whether to treat them.^5^

Endovenous laser thermal ablation is a safe option for treating VTs.^6^ When compared to phlebectomy, it reduces the number of incisions and the extent of hematoma and enables earlier return to work.^7^ With relation to ultrasound-guided sclerotherapy, it has the advantage of being local,^8^ avoiding distant effects.

**Figure 1:**
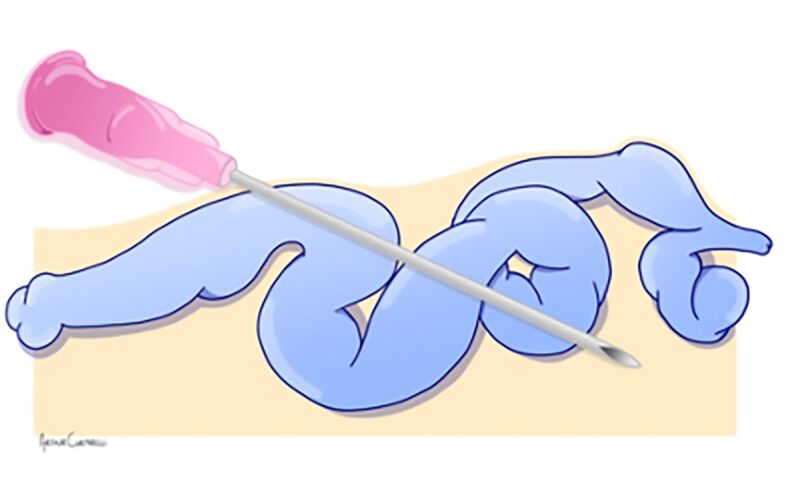
Puncture transfixing the venous walls.

**Figure 2:**
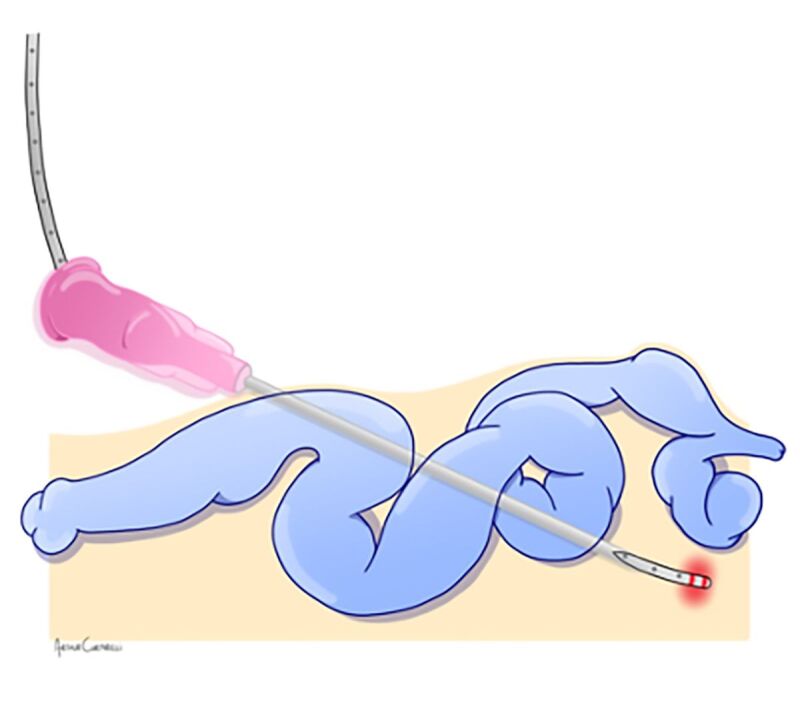
Advancing the optical fiber.

**Figure 3:**
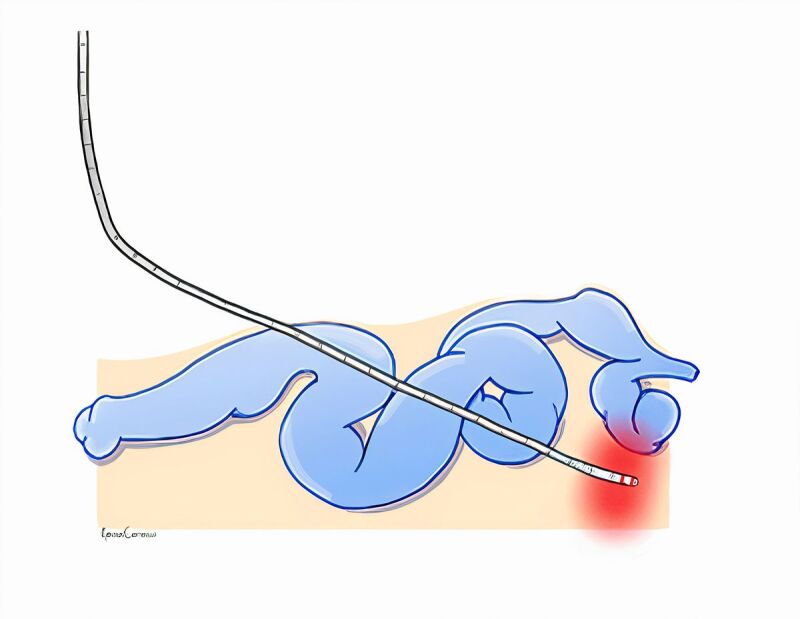
Perivenous and endovenous thermoablation.

**Figure 4:**
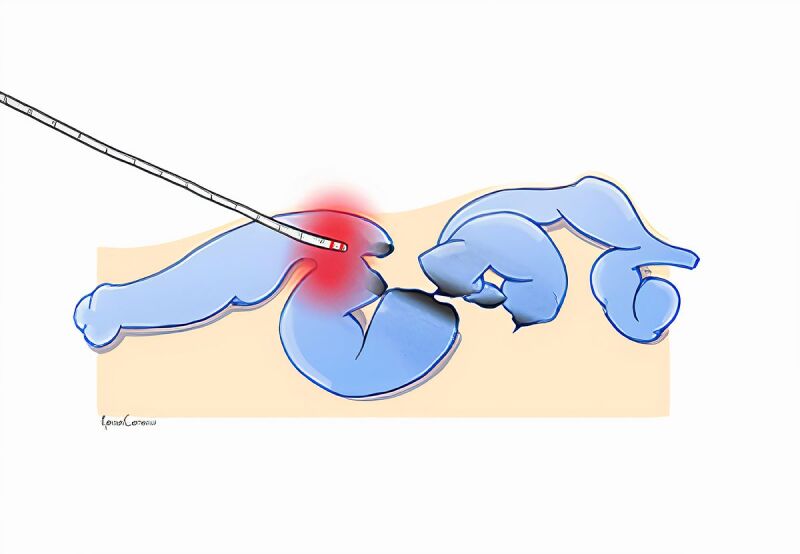
Endovenous thermoablation.

Publications about treatment of VTs using endolaser recommend multiple punctures, and, in particular, full insertion of introducer catheters into the vessel lumen.^9^ Another recommendation is that all accesses should be obtained before starting thermal ablation of the saphenous veins.^10^ This process takes time and can very often be unproductive, because some of the accesses are already no longer functional after ablation of the saphenous axis. However, we believe that it is possible to achieve adequate treatment by aiming not only to place the endolaser fiber within the venous lumen, but also using it outside the vessel, by puncturing and transfixing the vessel walls. This delivers the laser, both endovenously and perivenously, via all the layers of the vessel at the entry points and requires fewer punctures. This technique has been named transfixing endovenous thermal ablation (TEThA) (Figures 1 to 4).

This article reports a case of ambulatory treatment of the small saphenous vein (SSP) and VTs with endolaser thermoablation in a patient with PTS.

The TEThA technique was chosen in order to subject the patient to less invasive anesthesia, reduce the duration of the procedure, and, primarily, to reduce the volume of supplementary ultrasound-guided foam sclerotherapy, which in turn helps to mitigate the risk of thrombotic postoperative complications.

This project was approved by the Ethics Committee at the institution where it was conducted, under consolidated opinion number: 6.286.803.

## CASE DESCRIPTION

The patient was a 52-year-old woman with CEAP classification C3 and relapsed LL varicose veins after surgical treatment for varicose veins with phlebectomies around 6 years previously. During the first month after this procedure, she developed pain and edema in the right lower limb (RLL), and was diagnosed with acute venous thrombosis. Doppler ultrasonography showed presence of thrombi in the small saphenous, popliteal, gastrocnemius, soleus, and posterior tibial veins. She was treated with rivaroxaban for 6 months and thrombophilia was ruled out by investigation with laboratory tests.

The patient sought our service in March 2023 complaining of pain, tiredness,, cramps, and postural edema in the LL. She denied episodes of deep venous thrombosis prior to the varicose vein surgery, family history of thrombophilia, and other risk factors for thrombosis. She reported a family history of varicose veins and having undergone gastroplasty 15 years previously. Examination revealed large caliber varicose veins on the posterolateral aspect of the leg with diameters varying from 2 to 4 mm.

Venous Doppler ultrasonography of the RLL revealed ecographic signs of trabeculae in the popliteal vein and reflux in the gastrocnemius and small saphenous veins, extending from the lateral malleolus to the “J” point, with the following diameters: superior = 3.5 mm, medial = 2.6 mm, and inferior = 2.6 mm. Large caliber varicose veins were also observed on the posterolateral aspect of the leg with diameters varying from 2 to 4 mm.

The patient underwent ambulatory thermal ablation of the small saphenous vein and varicose tributary veins with the TEThA technique.

In this endolaser thermal ablation technique, accesses to the tributary veins were obtained sequentially after treatment of the trunk vein with punctures transfixing the tortuous varicose veins from the most distal segments (close to their runoff) to the most proximal (the origin of reflux in the saphenous vein). All procedures were conducted with generous tumescence with 0.08% lidocaine in saline solution to protect neighboring structures and, primarily, the skin. The wavelength employed is 1,470 nm and the laser is fired as the fiber is tractioned at a velocity of 1 mm/seg with power varying from 2 to 7 watts, using a maximum of 5 watts for 400 µm fibers and 7 watts for 600 µm fibers.

**Figure 5:**
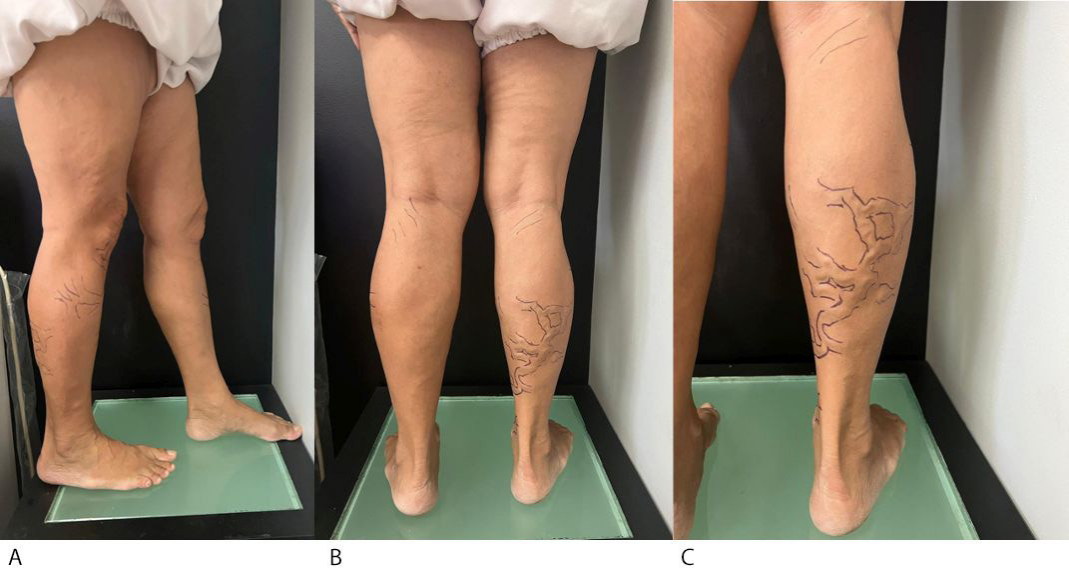
Preoperative photographic documentation of the lower limbs. (A) Lateral aspect; (B) Posterior aspect; (C) Preoperative marking of the varicose veins to be treated with the TEThA technique.

Immediately before the procedure, the varicose veins to be treated were marked on the skin (Figure 5C), which is a characteristic of this technique. A path is drawn adjacent to the varicose tributaries, enclosing their margins or placing the path of the varicose veins between parallel lines. This procedure is undertaken using a vein viewer and/or augmented reality device and complemented with Doppler ultrasonography in the supine position and is then documented photographically (Figures 5A and 5B). Under locoregional anesthesia with sedation with propofol (0.5 - 1 mg/kg), fentanyl (1 - 2 mcg/kg), and midazolam (0.05 - 0.2 mg/kg), the patient underwent thermal ablation of the proximal 2/3 of the right small saphenous vein (SSP) with endolaser at a wavelength of 1,470 nm, using a 400 nm radial fiber and with the energy level set at 8 watts of power. Only the proximal 2/3 were treated because of the well-known relationship between the sural nerve and the distal 1/3 of the SSP. Tributary varicose veins were punctured with a 16 G peripheral catheter using the TEThA technique. For this patient, four punctures were made, using energy levels varying from 2 to 4 watts, and after full thermoablation of the proximal segment of the SSP and the VTs, 3 mL of 2% polidocanol foam were administered, after using the Tessari technique to introduce the catheter, for chemical ablation of the distal third of the right SSP.

**Figure 6:**
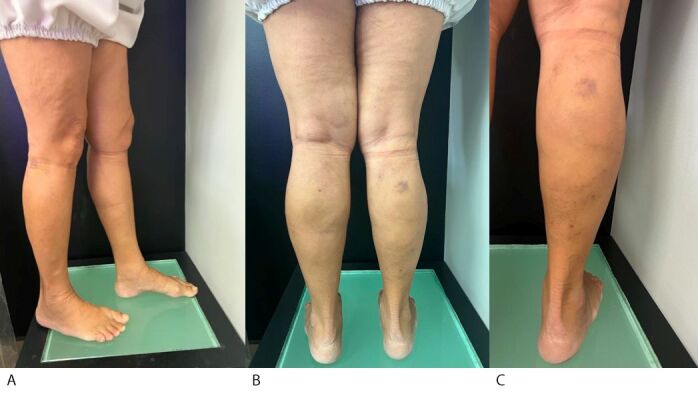
Postoperative photographic documentation of the lower limbs (after 12 days). (A) Lateral aspect; (B) Posterior aspect; (C) Postoperative appearance of varicose veins treated with the TEThA technique.

**Figure 7:**
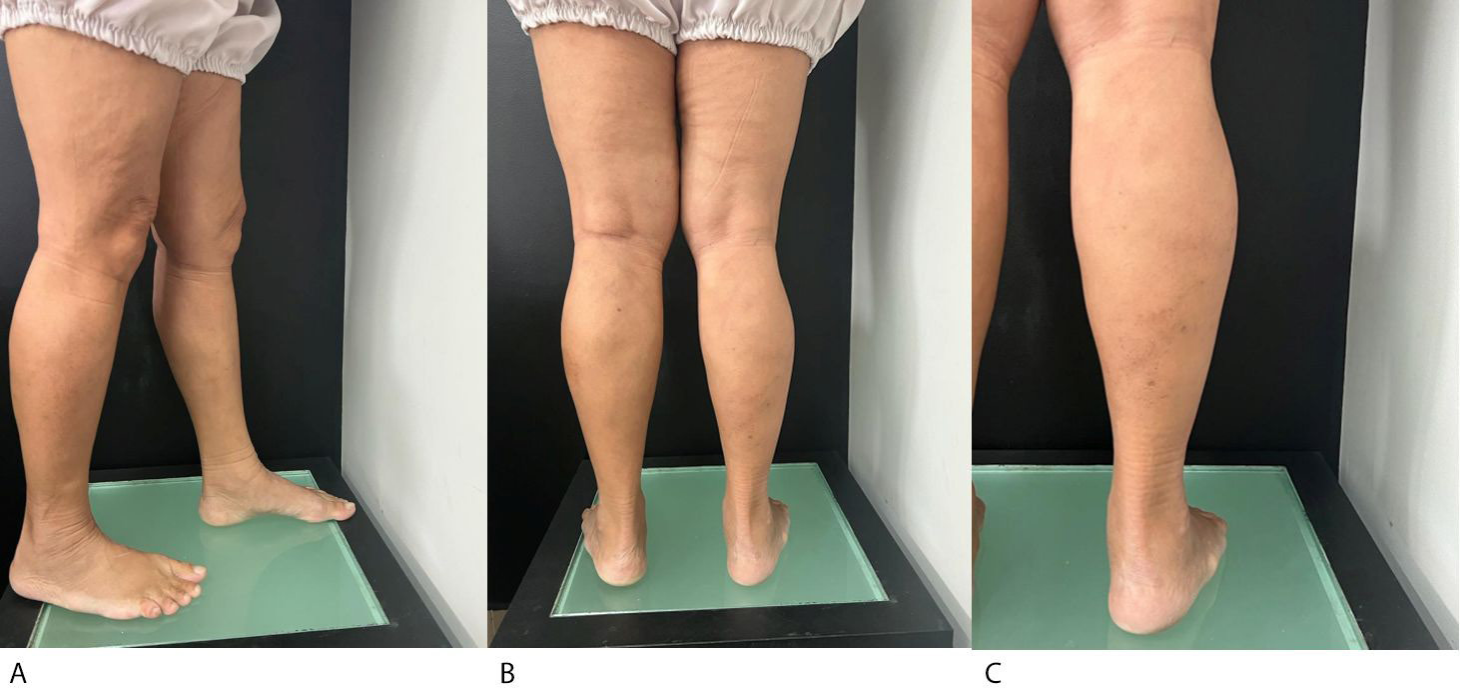
Postoperative photographic documentation of the lower limbs (after 30 days). (A) Lateral aspect; (B) Posterior aspect; (C) Postoperative appearance of varicose veins treated with the TEThA technique.

**Figure 8:**
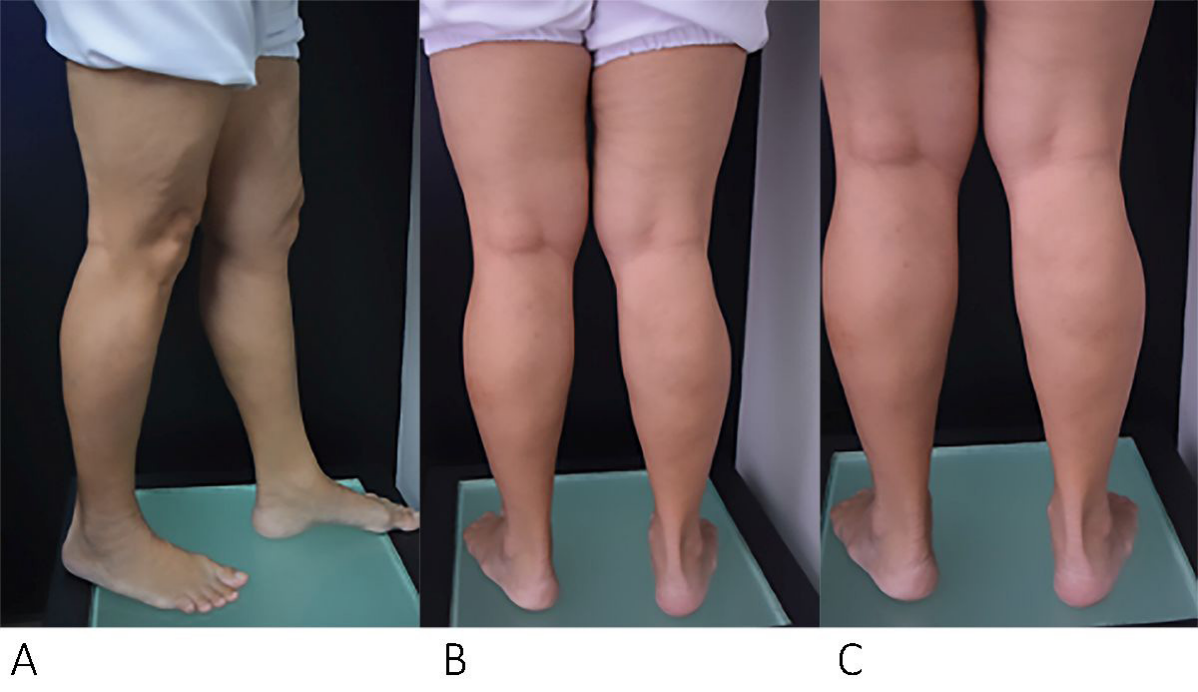
Postoperative photographic documentation of the lower limbs (after 60 days). (A) Lateral aspect; (B) Posterior aspect; (C) Postoperative appearance of varicose veins treated with the TEThA technique.

The patient was discharged soon after recovering from the anesthetic, around 2 hours after the procedure, walking, and wearing 35 mmHg compression hosiery for 24 hours. Her symptoms improved, she had no postoperative intercurrent conditions, and the volume of the varicose veins was reduced, as shown in the photographic record at 12 days (Figure 6), 30 days (Figure 7), and 60 days (Figure 8) after the procedure.

Control venous Doppler ultrasonography of the RLL at 30 days (Figure 7) showed absence of deep venous thrombosis, presence of occlusion of the small saphenous vein, and reduced volume of the varicose veins on the posterior aspect of the right leg, which were also occluded.

## DISCUSSION

We understand that phlebectomy is adequate for the majority of the spectrum of varicose veins, but, for treatment of VTs secondary to saphenous reflux, where thermal ablation of the axis is planned, extensive phlebectomy may be unnecessary, as demonstrated by staged treatment. In this series, only around 1/3 of patients requested adjunctive treatment of VTs 9 weeks after treatment of the saphenous axis.^5^

There are benefits to using the TEThA technique, in which the laser is already available to treat the VTs since it has been used to treat the saphenous axis, such as no need for incisions to perform phlebectomies, which involve a greater likelihood of deeper injury to the wall of the vessel when compared to foam sclerotherapy, which also mitigates the risk of sclerus formation.^11^ Another peculiarity is that authors who do not differentiate treatment of VTs with endovenous laser between those conducted concomitantly with the saphenous vein or not predict a need for a much higher number of punctures than are habitually needed for TEThA,^12^ which is probably because they do not take advantage of the hemodynamic remodeling caused by treatment of the reflux axis itself.

In the literature, the majority of authors only use endovenous laser thermal ablation for reflux in saphenous veins and do not use it for tributary varicose veins. Although resolution of symptoms and significant improvement in the appearance of the leg are generally observed after treatment with endolaser, adjuvant procedures with sclerotherapy or phlebectomy are very often needed. However, it is known that endolasers can occlude both the venous trunk and tributary varicose veins with efficacy and low morbidity.^11^

In contrast with other techniques for treatment of VTs that are described in publications, in which all of the punctures in each sector are performed before starting thermal ablation and total endovenous ablation,^10^ in TEThA, ablation is performed progressively, one by one, and energy is delivered both inside and outside of the vein (endovenously and perivenously). While it does require use of certain devices and there is a learning curve, the curve is rapid.^13^

The disadvantages, when compared to foam sclerotherapy as adjuvant treatment for VTs, are the increased duration of the procedure and the higher risk of reintervention.^9^ Both can be justified by the esthetic improvements, the reduced volume of sclerosing agent needed to treat more extensive cases, and even by the possibility of not using sclerotherapy in specific cases. It is notable that there are not yet any studies comparing endovenous laser and phlebectomy for treatment of VTs, but it is not expected that the same difference will be observed.

In conclusion, combined endovenous and perivenous treatment of VTs using the TEThA technique described in this article was demonstrated to be a new, less invasive, treatment option. Moreover, the treatment described resolved the symptoms and did not provoke a repeat thrombotic event.
