# Supplementary material for: Treatment of the small saphenous vein and tributary veins with endolaser associated with ultrasound-guided foam in a patient with post-thrombotic syndrome: presenting the TEThA technique
Source: J Vasc Bras. 2024 Nov 29;23:e20230142. doi: 10.1590/1677-5449.202301422 (PMC11614099; doi:10.1590/1677-5449.202301422)
Supplement: Translation

# Translation: Tratamento de veia safena parva e de veias tributárias com *endolaser* associado a espuma ecoguiada em paciente com síndrome pós-trombótica: apresentando a técnica TEThA

**DOI:** 10.1590/1677-5449.202301421

**Authors:** Nara Medeiros Cunha de Melo Vasconcelos, Harue Santiago Kumakura, Marcelo Halfen Grill, Marília de Castro e Silva

**Affiliations:** 1 Hospital Dia Anjos Vasculares, Natal, RN, Brasil.; 2 Centro de Estudos Hiroshi Miyake, São Paulo, SP, Brasil.

**Keywords:** varizes, veia safena, insuficiência venosa crônica, *laser* endovenoso, estética

## Abstract

A doença venosa crônica dos membros inferiores possui alta prevalência, com termoablação endovenosa como técnica de escolha para o tratamento de veias safenas insuficientes. Porém, ainda há controvérsia sobre a melhor conduta para as veias tributárias varicosas. Este artigo relata um caso de tratamento ambulatorial de refluxo de veia safena parva e de veias tributárias em paciente do sexo feminino, de 52 anos, com síndrome pós-trombótica e queixa de dor e edema em membro inferior direito. Realizamos a técnica transfixing endovenous thermal ablation (TEThA) com termoablação da veia safena parva e de veias varicosas, combinada com espuma ecoguiada de polidocanol a 2%. Após 30 dias, no eco-Doppler de controle, havia oclusão de veia safena parva e ausência de sinais ecográficos de varizes em face posterior de perna direita e de trombose. O tratamento combinado endovenoso e perivenoso das varizes dos membros inferiores tem se mostrado seguro, rápido e eficaz.

## INTRODUÇÃO

A doença venosa crônica (DVC) dos membros inferiores (MMII) possui alta prevalência^1^ . Quando não tratada, 22% daqueles pacientes classificados como CEAP C2^2^ vão progredir para úlcera venosa em 6 anos^1^ . A síndrome pós-trombótica (SPT) é um conjunto de sintomas e sinais de DVC causado por comprometimento do fluxo venoso como resultado de obstrução venosa profunda e/ou refluxo após trombose venosa profunda. Os sinais típicos são dor, varizes, edema e alterações cutâneas, incluindo úlcera venosa, em MMII. Esta síndrome ocorre em 20 a 50% dos pacientes com TVP, dos quais 5 a 10% desenvolvem SPT grave^3^ .

Enquanto a termoablação endovenosa é considerada a técnica de escolha para o tratamento de veias safenas insuficientes, ainda há controvérsia sobre a melhor conduta para as tributárias varicosas (TVs)^4^ . Apesar de o tratamento concomitante das TVs através da flebectomia manter sua indicação^4^ , existe uma tendência por buscar técnicas menos invasivas, como a escleroterapia química ou térmica, e até mesmo aguardar, após a ablação das veias safenas insuficientes, a repercussão da diminuição da pressão venosa no remodelamento das TVs para decidir o tratamento ou não das mesmas^5^ .

A termoablação com *laser* endovenoso é uma alternativa segura para o tratamento das TVs^6^ . Quando comparada à flebectomia, essa reduz o número de cortes e hematomas, além de antecipar o retorno ao trabalho^7^ . Em relação à escleroterapia ecoguiada, tem a vantagem de ser local^8^ , evitando o efeito à distância.

É preconizado, nas publicações sobre o tratamento de TVs com o *endolaser*, múltiplas punções e, principalmente, a colocação por completo de cateteres introdutores dentro da luz do vaso^9^ . Outra orientação existente é que todos os acessos sejam obtidos antes da termoablação das veias safenas^10^ . Esse processo demanda tempo e muitas vezes se mostra improdutivo, pois parte dos acessos já não se encontram funcionais após a ablação do eixo safênico. Todavia, acreditamos que é possível fazer um tratamento adequado objetivando não só a locação da fibra do *endolaser* dentro da luz venosa, mas também fora, através da punção com a transfixação das paredes dos vasos. Isso resulta na entrega do *laser*, tanto endovenosamente quanto perivenosamente, através de todas as camadas do vaso nos pontos de entrada e necessitando de um número menor de punções. Essa técnica foi nomeada pelo acrônimo em inglês *transfixing endovenous thermal ablation* (TEThA) (Figuras 1 a 4).

**Figura 1:** Punção com transfixação das paredes venosas.

**Figura 2:** Passagem da fibra ótica.

**Figura 3:** Termoablação peri e endovenosa.

**Figura 4:** Termoablação endovenosa.

Este artigo relata um caso de tratamento ambulatorial com termoablação com *endolaser* de veia safena parva (VSP) e de TVs em paciente com SPT. Foi decidido pela utilização da técnica TEThA com objetivo de submeter a paciente a uma anestesia menos invasiva, diminuir o tempo do procedimento e, principalmente, reduzir o volume de escleroterapia com espuma ecoguiada complementar associada, o que, por sua vez, ajuda a mitigar o risco de complicação trombótica pós-operatória.

O presente trabalho foi aprovado pelo Comitê de Ética da instituição onde foi realizado, tendo o seguinte número do parecer consubstanciado: 6.286.803.

## DESCRIÇÃO DO CASO

A paciente era do sexo feminino, tinha 52 anos de idade e apresentava classificação CEAP C3, com quadro de varizes recidivadas em MMII após tratamento cirúrgico de varizes com flebectomias há cerca de 6 anos. No decorrer do primeiro mês após esse procedimento, evoluiu com dor e edema no membro inferior direito (MID), sendo diagnosticada com trombose venosa aguda. O eco-Doppler, por sua vez, evidenciou a presença de trombos nas veias safena parva, poplítea, gastrocnêmias, soleares, e tibiais posteriores. Foi realizado tratamento com rivaroxabana por 6 meses e descartado diagnóstico de trombofilia por investigação com exames laboratoriais.

A paciente procurou nosso serviço em março de 2023 com queixa de dor, cansaço, câimbras e edema postural em MMII. Negava episódios de trombose venosa profunda prévios à cirurgia de varizes, história familiar de trombofilia, e outros fatores de risco para trombose. Relatava história familiar de varizes e realização de gastroplastia há 15 anos. Foi constatada também a presença de varizes calibrosas em perna na face póstero-lateral com diâmetros variando de 2 a 4 mm.

Seu eco-Doppler venoso do MID evidenciou sinais ecográficos de trabéculas em veia poplítea e refluxo em veia gastrocnêmia e em veia safena parva, com extensão do maléolo lateral ao ponto “J”, que apresentava os seguintes diâmetros: superior = 3,5 mm, medial = 2,6 mm e inferior = 2,6 mm. Foi constatada também a presença de varizes calibrosas em perna na face póstero-lateral com diâmetros variando de 2 a 4 mm.

A paciente foi submetida a termoablação da veia safena parva e das veias tributarias varicosas em regime ambulatorial com a técnica de TEThA.

Nesta técnica de termoablação com endolaser, os acessos das veias tributárias são obtidos de forma sequenciada após o tratamento da veia troncular através de punções transfixando as tortuosidades das varizes, dos trechos mais distais (próximo ao desague) para proximais (origem do refluxo na veia safena). Tudo sob generosa tumescência com 0,08% de lidocaína em solução salina para proteger as estruturas vizinhas e, principalmente, a pele. O comprimento de onda preconizado é o de 1.470 nm, e o *laser* é disparado à medida em que a fibra é tracionada a uma velocidade de 1 mm/seg e potência variando de 2 a 7 watts, utilizando-se 5 watts no máximo para as fibras de 400 µm e 7 watts para as fibras de 600 µm.

Imediatamente antes do procedimento, foi realizada a marcação pré-operatória das varizes a serem tratadas (Figura 5C), o que é característica desta técnica, onde é preconizado o desenho de um traçado adjacente às tributárias varicosas, circundando os seus bordos ou posicionando o enovelado das varizes entre linhas paralelas. Isso tudo sob o auxílio de fleboscópio e/ou dispositivo de realidade aumentada e complementação com eco-Doppler em posição supina, sendo então feita fotodocumentação (Figura 5A e 5B). Sob anestesia locorregional com sedação com propofol (0,5 - 1 mg/kg), fentanil (1 - 2 mcg/kg) e midazolam (0,05 - 0,2 mg/kg), a paciente foi submetida a termoablação de 2/3 proximais da veia safena parva (VSP) direita com *endolaser* de comprimento de onda 1.470 nm com fibra radial de 400 micras e energia de 8 watts de potência. Somente os 2/3 proximais foram tratados devido a conhecida relação do nervo sural com a VSP no seu 1/3 distal. Foram realizadas punções das tributárias varicosas com cateter periférico 16 G através da técnica TEThA. Nessa paciente, foram realizadas cerca de 4 punções com energia variando de 2 a 4 watts, e após a termoablação do trecho proximal da VSP e das TVs, realizou-se a aplicação de 3 mL de espuma com polidocanol a 2%, utilizando a técnica de Tessari pelo cateter introdutor para a ablação química do terço distal da VSP direita.

**Figura 5:** Fotodocumentação pré-operatória dos membros inferiores. (A) Face lateral; (B) Face posterior; (C) Marcação pré-operatória das varizes submetidas a técnica TEThA.

A paciente recebeu alta logo após a recuperação anestésica, cerca de 2 horas depois do procedimento, deambulando e utilizando meia de compressão 35 mmHg por 24 horas. Evoluiu com melhora de sintomas e sem intercorrências pós-operatórias, além da redução do volume das veias varicosas, evidenciada na fotodocumentação após 12 dias (Figura 6), 30 dias (Figura 7), e 60 dias (Figura 8) do procedimento.

**Figura 6:** Fotodocumentação pós-operatória dos membros inferiores (após 12 dias). (A) Face lateral; (B) Face posterior; (C) Aspecto pós-operatório das varizes submetidas a técnica TEThA.

**Figura 7:** Fotodocumentação pós-operatória dos membros inferiores (após 30 dias). (A) Face lateral; (B) Face posterior; (C) Aspecto pós-operatório das varizes submetidas a técnica TEThA.

**Figura 8:** Fotodocumentação pós-operatória dos membros inferiores (após 60 dias). (A) Face lateral; (B) Face posterior; (C) Aspecto pós-operatório das varizes submetidas a técnica TEThA.

O eco-Doppler venoso do MID de controle após 30 dias (Figura 7) evidenciou ausência de trombose venosa profunda, presença de oclusão de veia safena parva e diminuição do volume das varizes em face posterior de perna direita que também se encontravam ocluídas.

## DISCUSSÃO

Entendemos que a flebectomia é adequada na maioria dos espectros das varizes, porém, no tratamento de TVs secundárias ao refluxo de safena, onde a termoablação do eixo está planejada, a realização de flebectomia extensa pode ser desnecessária, como foi demonstrado no tratamento estagiado. Neste, somente cerca de 1/3 dos pacientes solicitam a complementação do tratamento das TVs após 9 semanas do tratamento do eixo safênico^5^ .

Na técnica TEThA, onde o *laser* já está disponível para o tratamento das TVs por ter sido usado no tratamento do eixo safênico, existem benefícios, como a ausência das incisões das flebectomias, que têm maior possibilidade de lesão mais profunda na parede do vaso quando comparada a escleroterapia com espuma, mitigando também o risco de formação de esclerus^11^ .Outra peculiaridade é que os autores que não diferenciam o tratamento com *laser* endovenoso das TVs, daquelas que são feitas concomitante ou não com a veia safena, preconizam a necessidade de quantidade de punções muito maiores do que é habitualmente usado na TEThA^12^ , provavelmente não tirando proveito da remodelação hemodinâmica causada apenas pelo tratamento do eixo de refluxo.

Na literatura, a maioria dos autores utiliza apenas a termoablação com *laser* endovenoso para o refluxo das veias safenas, e não para as varicosidades tributárias. Embora a resolução sintomática e a melhora significativa da aparência da perna geralmente sejam observadas após tratamento com *endolaser*, muitas vezes são necessários procedimentos complementares, como escleroterapia ou flebectomia. Contudo, já sabe-se que o *endolaser* oclui tanto o tronco venoso quanto as varicosidades tributárias com eficácia e baixa morbidade^11^ .

Diferentemente de outras técnicas publicadas preconizadas para o tratamento das TVs, onde todas as punções de cada setor são realizadas antes do início da termoablação e ablação totalmente endovenosa^10^ , na TEThA a ablação ocorre de forma progressiva, uma a uma, com a energia sendo entregue tanto dentro como fora da veia (endo e perivenosa).Embora suponha a utilização de alguns dispositivos e uma curva de aprendizagem, esta última é rápida^13^ .

As desvantagens, quando comparada à escleroterapia com espuma como tratamento adjuvante das TVs, são o aumento da duração do procedimento e o maior risco de reintervenção^9^ . Ambas podem ser justificadas pela melhora estética, pela diminuição do volume de esclerosante necessário para o tratamento de casos mais extensos e, até mesmo, pela possibilidade de não fazer uso de escleroterapia em casos específicos. Nota-se ainda que não existem estudos comparando o tratamento de TVs entre *laser* endovenoso e flebectomia, mas não se espera constatar a mesma diferença.

Concluindo, o tratamento combinado endovenoso e perivenoso das TVs, através da técnica TEThA descrita neste artigo, tem se mostrado como uma nova opção terapêutica menos invasiva. E, diante do exposto, o tratamento realizado foi resolutivo e não provocou novo evento trombótico.
